# Functional characterisation of peroxisomal β-oxidation disorders in fibroblasts using lipidomics

**DOI:** 10.1007/s10545-017-0076-9

**Published:** 2017-08-28

**Authors:** Katharina Herzog, Mia L. Pras-Raves, Sacha Ferdinandusse, Martin A. T. Vervaart, Angela C. M. Luyf, Antoine H. C. van Kampen, Ronald J. A. Wanders, Hans R. Waterham, Frédéric M. Vaz

**Affiliations:** 1 Laboratory Genetic Metabolic Diseases, Departments of Clinical Chemistry and Pediatrics, University of Amsterdam, Meibergdreef 9, Amsterdam, 1105 AZ The Netherlands; 2 Department of Clinical Epidemiology, Biostatistics, and Bioinformatics, Academic Medical Center, University of Amsterdam, Meibergdreef 9, Amsterdam, 1105 AZ The Netherlands; 3 Biosystems Data Analysis, Swammerdam Institute for Life Sciences, University of Amsterdam, Science Park 904, Amsterdam, 1098 XH The Netherlands

**Keywords:** Peroxisomes, β-oxidation, Lipidomics, Phospholipids, Very long-chain fatty acids, Plasmalogens

## Abstract

Peroxisomes play an important role in a variety of metabolic pathways, including the α- and β-oxidation of fatty acids, and the biosynthesis of ether phospholipids. Single peroxisomal enzyme deficiencies (PEDs) are a group of peroxisomal disorders in which either a peroxisomal matrix enzyme or a peroxisomal membrane transporter protein is deficient. To investigate the functional consequences of specific enzyme deficiencies on the lipidome, we performed lipidomics using cultured skin fibroblasts with different defects in the β-oxidation of very long-chain fatty acids, including ABCD1- (ALD), acyl-CoA oxidase 1 (ACOX1)-, D-bifunctional protein (DBP)-, and acyl-CoA binding domain containing protein 5 (ACBD5)-deficient cell lines. Ultra-high performance liquid chromatography coupled with high-resolution mass spectrometry revealed characteristic changes in the phospholipid composition in fibroblasts with different fatty acid β-oxidation defects. Remarkably, we found that ether phospholipids, including plasmalogens, were decreased. We defined specific phospholipid ratios reflecting the different enzyme defects, which can be used to discriminate the PED fibroblasts from healthy control cells.

**Electronic supplementary material:**

The online version of this article (doi:10.1007/s10545-017-0076-9) contains supplementary material, which is available to authorized users.

## Introduction

**Fig. 1:**
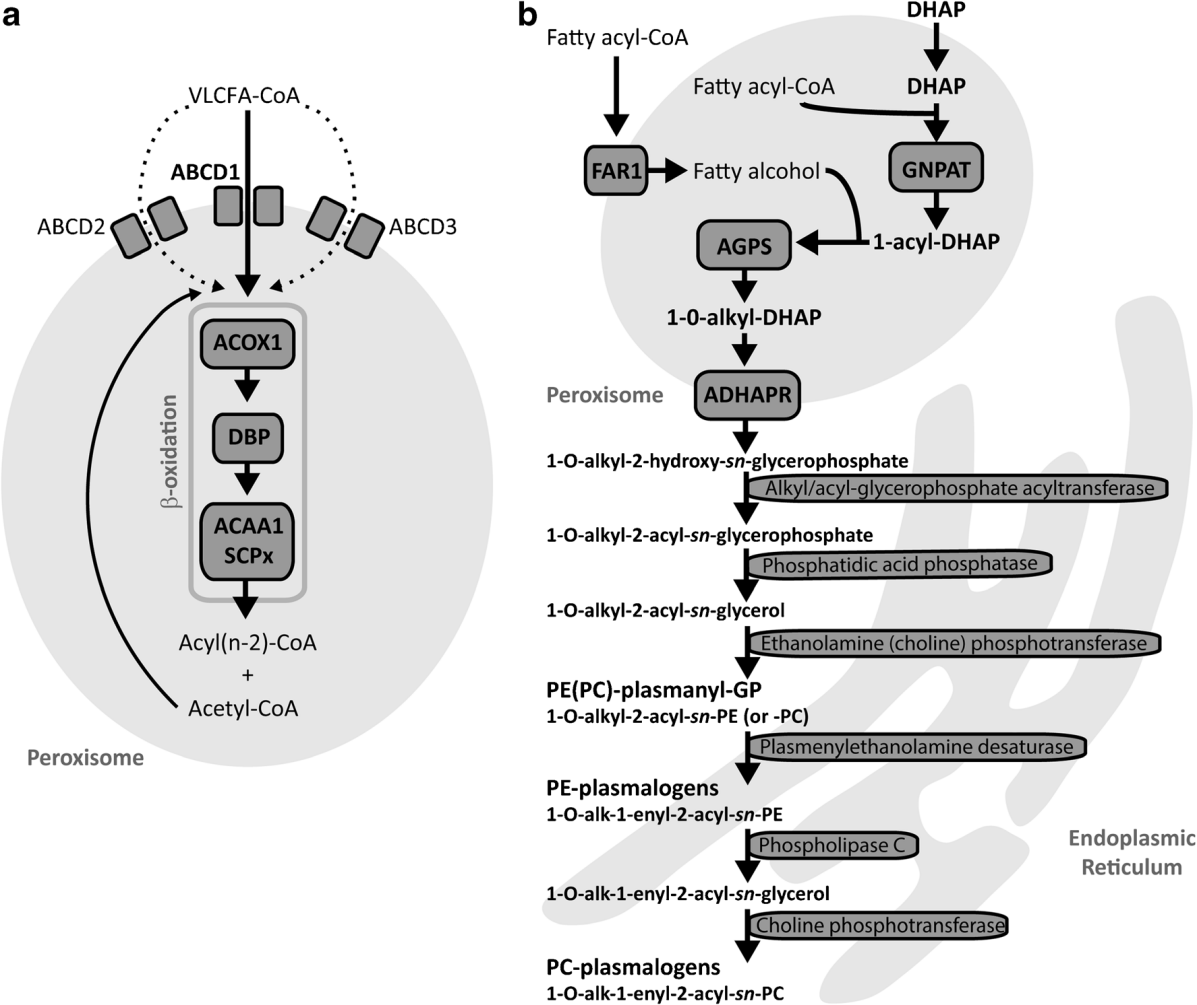
Schematic overview of peroxisomal β-oxidation and ether phospholipid biosynthesis. a) Metabolism of very long-chain fatty acids (VLCFAs), in particular the enzymes and proteins involved in β-oxidation are shown. Saturated and unsaturated VLCFAs enter the peroxisome as coenzyme A (CoA) esters via the ABC transporter D1 (ABCD1, ALD protein (ALDP)), and alternatively via the ABC transporters D2 (ABCD2), and D3 (ABCD3), respectively, and subsequently undergo one or several rounds of β-oxidation. Each round comprises a cycle of dehydrogenation, hydration, dehydrogenation, and thiolytic cleavage, and results in the production of a 2-carbon-shortened fatty acid and acetyl-CoA. The enzymes involved in β-oxidation of VLCFAs are acyl-CoA oxidase 1 (ACOX1), D-bifunctional protein (DBP), and 3-ketoacyl-CoA thiolase (ACAA1) or sterol carrier protein X (SCPx). b) Biosynthesis of ether phospholipids. The first two reactions of ether phospholipid biosynthesis take place in the peroxisome. First, dihydroxyacetone phosphate (DHAP) is acylated with a fatty acyl-CoA at the sn-1 position by the enzyme glyceronephosphate O-acyltransferase (GNPAT). Next, alkylglycerone phosphate synthase (AGPS) catalyses the exchange of the fatty acyl group of 1-acyl-DHAP for a fatty alcohol. The fatty alcohol is converted from a fatty acyl-CoA outside of the peroxisome and subsequently imported into the organelle, reactions that are performed by fatty acyl reductase 1 (FAR1). The produced 1-O-alkyl-DHAP is then converted to 1-O-alkyl-2-hydroxy-sn-glycerophosphate by the enzyme acyl/alkyl-DHAP reductase (ADHAPR), a process which can occur both in the peroxisome and in the endoplasmic reticulum (ER). The following reactions take place in the ER. The intermediate 1-O-alkyl-2-acyl-sn-glycerol is formed by alkyl/acyl-glycerophosphate transferase, which exchanges the hydroxy group at the sn-2 position of the substrate for an acyl-group. Subsequently, phosphatidic acid phosphatase removes the phosphate group to form 1-O-alkyl-2-acyl-sn-glycerol. Next, PE- and PC-plasmanyl glycerophosphate (GP) species are formed by ethanolamine and choline phosphotranferase, respectively, by incorporation of CDP-ethanolamine and -choline. The alkyl group of PE-plasmanyl-GP is then dehydrogenated by plasmenylethanolamine desaturase to form PE-plasmenyl-GP (PE-plasmalogens). Finally, since no plasmenylcholine desaturase exits, the head group of PE-plasmalogen species is hydrolysed by phospholipase C and subsequently CDP-choline is incorporated by choline phosphotransferase to form PC-plasmalogens

Peroxisomes are ubiquitous cell organelles that play an important role in various metabolic pathways of eukaryotes. In humans, they are involved in both catabolic and anabolic metabolic processes (Wanders and Waterham 2006), including the α- and β-oxidation of a variety of fatty acids, the biosynthesis of ether phospholipids and the detoxification of glyoxylate and reactive oxygen species such as hydrogen peroxide (Van Veldhoven 2010; Waterham et al 2016) (Fig. 1). Although both peroxisomes and mitochondria are involved in the breakdown of fatty acids, oxidation of very long-chain fatty acids (VLCFAs, such as C24:0 and C26:0), branched-chain fatty acids (phytanic and pristanic acid), and bile acid precursors di- and trihydroxycholestanoic acid (DHCA and THCA) only occurs in peroxisomes (Waterham et al 2016). VLCFAs enter the peroxisome as CoA-esters via the half-transporters ABCD1, and, to a lesser extent, ABCD2 and ABCD3 (Fig. 1), and are degraded by β-oxidation which involves four subsequent enzyme steps, including desaturation, hydration, dehydrogenation and thiolytic cleavage (Van Veldhoven 2010; Van Roermund et al 2011; Wiesinger et al 2013). The peroxisomal enzymes acyl-CoA oxidase 1 (ACOX1), D-bifunctional protein (DBP), and the peroxisomal thiolases 3-oxoacyl-CoA thiolase and sterol carrier protein X, catalyse this sequence of reactions (Van Veldhoven 2010) (Fig. 1A). Per cycle of β-oxidation, one acetyl-CoA and an acyl-CoA shortened by two carbon atoms are produced. The latter can be subject to another round of peroxisomal β-oxidation, or transported to mitochondria for further breakdown (Wanders and Waterham 2006).

Defects in peroxisome function result in a large number of metabolic disorders, with a broad spectrum of disease severity. These peroxisomal disorders can be subdivided into the peroxisome biogenesis disorders (PBDs) and the single peroxisomal enzyme deficiencies (PEDs) (Waterham and Ebberink 2012). Because the biogenesis of peroxisomes is affected in PBDs, multiple metabolic pathways are deficient, whereas in PED the peroxisomes are correctly assembled and functional, but due to the deficiency of a single peroxisomal enzyme/transporter protein typically one specific pathway is affected. Based on which metabolic pathway is affected, the PEDs can be divided into different subgroups (Wanders and Waterham 2006; Poll-The and Gärtner 2012). PEDs affecting the β-oxidation of VLCFAs are X-linked adrenoleukodystrophy (ALD), ACOX1-, DBP-, and Acyl-CoA binding domain containing protein 5 (ACBD5) deficiency.

ALD (OMIM #300100) is the most frequently occurring peroxisomal disorder, and is caused by a defect in the *ABCD1* gene, which encodes a transmembrane transporter protein that imports straight chain VLCFA-CoA esters into the peroxisome (Kemp et al 2012) (Fig. 1). Biochemically, patients with ALD have elevated levels of straight-chain VLCFAs in plasma and tissues, and the rate of VLCFA β-oxidation in cells is reduced (Poll-The and Gärtner 2012; Waterham et al 2016). Clinical features of ALD can range from a non-inflammatory axonopathy to severe cognitive and neurologic disability with progressive white matter demyelination (Kemp et al 2012).

ACOX1 deficiency (OMIM #264470) is a peroxisomal enzyme defect that results in impaired β-oxidation of VLCFAs. ACOX1 catalyses the first step of peroxisomal β-oxidation where it oxidises straight-chain fatty acids, such as VLCFAs and polyunsaturated fatty acids (Ferdinandusse et al 2007) (Fig. 1). Biochemically, patients with ACOX1 deficiency have elevated levels of straight-chain VLCFAs in plasma and tissues, whereas the levels of branched-chain fatty acids including phytanic acid and the bile acid intermediates are normal (Ferdinandusse et al 2007). Common clinical symptoms include muscular hypotonia and seizures, starting in the neonatal period (Poll-The and Gärtner 2012).

DBP catalyses the second and third step of the peroxisomal β-oxidation cycle, and its proper functioning is crucial for the fatty acid breakdown in peroxisomes (Ferdinandusse et al 2006; Van Veldhoven 2010). In plasma and tissue samples from patients with DBP deficiency (OMIM #261515), increased levels of a number of metabolites can be found, including VLCFAs, THCA, DHCA, and pristanic acid (Ferdinandusse et al 2006). Clinically, patients with DBP deficiency commonly present with severe neurological symptoms, including neonatal hypotonia, seizures and a short life expectancy (Poll-The and Gärtner 2012).

ACBD5 deficiency has recently been described as a new peroxisomal disorder (Ferdinandusse et al 2016; Yagita et al 2017). ACBD5 is a peroxisomal membrane protein with a cytosolic acyl-CoA binding domain, and is postulated to facilitate transport of VLCFA-CoAs into the peroxisome (Ferdinandusse et al 2016). The main biochemical feature of ACBD5 deficiency is the accumulation of VLCFAs. Only a few patients with ACBD5 deficiency have been diagnosed to date, who presented with progressive leukodystrophy, ataxia, and retinal dystrophy (Abu-Safieh et al 2013; Ferdinandusse et al 2016; Yagita et al 2017).

In this study, we used ultra-high performance liquid chromatography coupled with high-resolution mass spectrometry (UPLC-HRMS) to gain more insight into the pathophysiology and the functional consequences of PEDs affecting peroxisomal β-oxidation on the lipidome of skin fibroblasts. We found characteristic changes in the phospholipid profiles of the different PEDs, which reflect their heterogeneity and highlight the different roles of the affected enzymes and transporter proteins in peroxisomal metabolism. Remarkably, we also found a decrease in selected ether phospholipid species, including plasmalogens, in all the PED cells. Our study revealed specific and discriminative phospholipid ratios that reflect the defects of the PED cells when compared to healthy control fibroblasts.

## Materials and methods

### Cultured skin fibroblasts

All cell lines were anonymised. We used primary skin fibroblast cell lines from seven healthy controls, seven patients with ACOX1 deficiency, six patients with DBP deficiency, seven ALD patients (i.e. ABCD1 deficiency), and one patient with a deficiency of ACBD5. Fibroblasts were cultured in 162-cm^2^ flasks in Ham’s F-10 Medium with L-glutamine, supplemented with 10% foetal calf serum (Invitrogen, Carlsbad, CA, USA), 25 mM Hepes, 100 U/mL penicillin, 100 μg/mL streptomycin, and 250 μg/mL amphotericin in a humidified atmosphere of 5% CO2 at 37 °C. All cells were cultured under the same condition using the same batch of medium and additives to prevent medium-induced changes in lipid composition. We harvested the cells by trypsinisation (0.5% trypsin-EDTA, Invitrogen) after they reached confluency, and washed once with phosphate-buffered saline and twice with 0.9% NaCl, followed by centrifugation at 4 °C (16,100 x g for 5 min) to obtain cell pellets. Cell pellets were stored at −80 °C until analysis.

### Biochemical analyses, phospholipid extraction procedure, and UPLC-HRMS

VLCFAs were measured as described previously (Vreken et al 1998). Lipidomic analysis was performed as described previously (Herzog et al 2016).

### Bioinformatics and statistics

The dataset was processed using an in-house developed metabolomics pipeline (Herzog et al 2016). Total phospholipid levels as presented in this paper are defined as the summation of the abundance, relative to the corresponding internal standard, of all identified phospholipid species of the same class, by assuming identical response with respect to the internal standard. Data in figures are presented as mean ± SD, and a Students t-test or one-way ANOVA with post-hoc correction according to the method of Benjamini and Hochberg (q-values) was used for statistical comparison between the groups. Partial least squares regression discriminant analysis (PLS-DA) with the extraction of variable importance in projection (VIP) scores was performed using the R package ‘ropls’ (Thévenot et al 2015).

## Results

### Changes in specific phospholipid classes in peroxisomal β-oxidation-deficient cells

Using a lipidomics approach, we investigated the phospholipid profiles in cultured control fibroblasts, and PED fibroblasts affecting fatty acid β-oxidation, including ABCD1- (ALD), ACBD5-, ACOX1-, and DBP-deficient cells. We annotated 936 distinct phospholipid species that were present in all samples (Suppl. Table A). Phospholipids identified in our analysis are denoted as C(XX:Y), where XX denotes the total number of carbon atoms and Y the total number of double bonds in the fatty-acyl side chains.

**Fig. 2:**
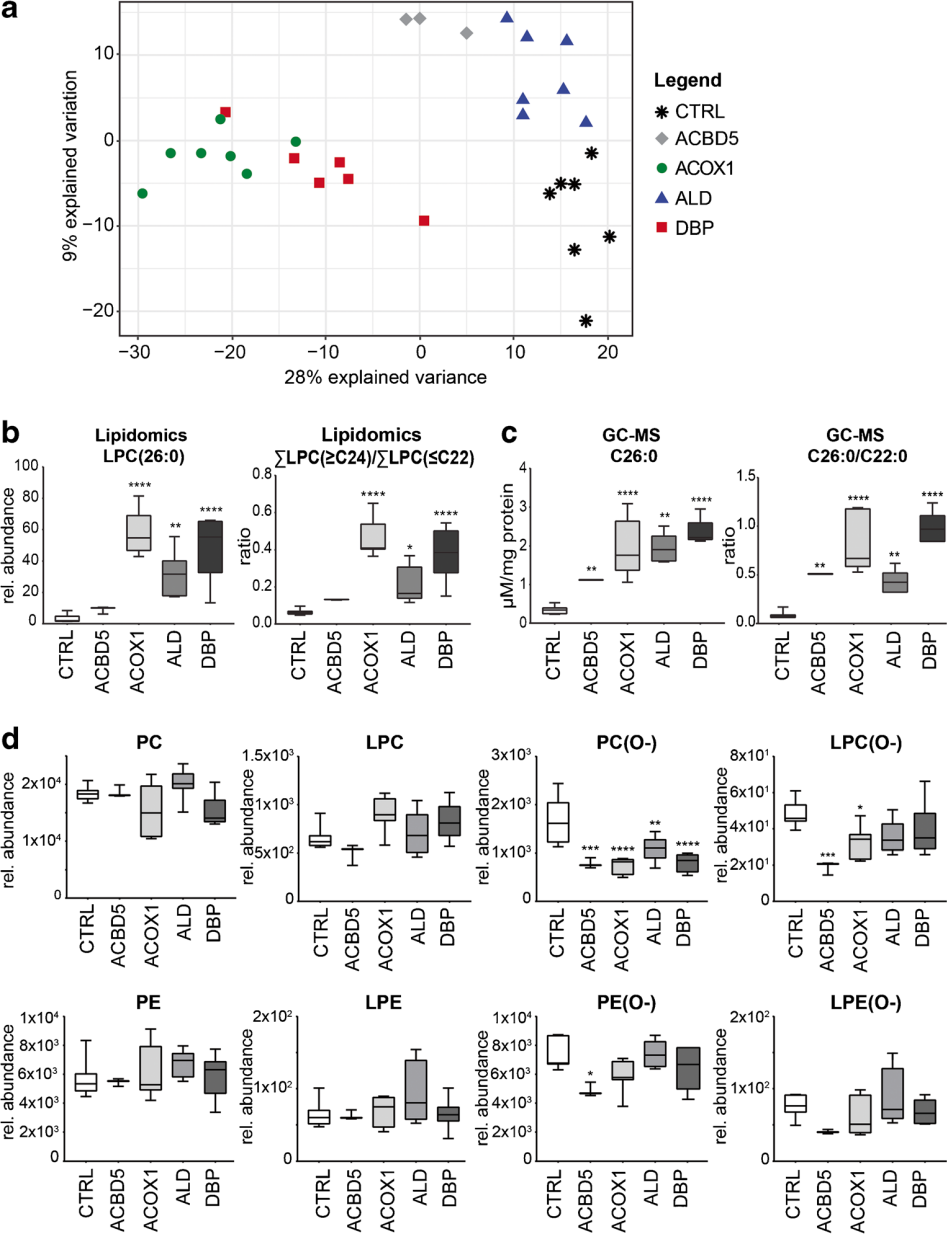
Phospholipid profiles of PED patient fibroblasts. a) Multivariate model of variations phospholipid composition using PLS-DA. Score plot of the model in the plane of the first (X-variate) and second component (Y-variate). The percentage of variance explained by the components is indicated in parentheses. The percentage of predictor variance explained by the full model (RX2) is 0.285; the percentage of response variance (RY2) is 0.242. The predictive performance of the model estimated by cross-validation (Q2Y) is 0.225. b) Left panel: relative abundance of LPC(26:0), right panel: ratio of LPC species containing VLCFAs (≥C24) over LCFAs (≤C22), determined by lipidomics. c) Left panel: total C26:0 levels, and right panel: ratio of total C26:0 over C22:0 levels, measured by derived from GC-MS measurement of total VLCFAs. d) Total phospholipid levels, sorted by phospholipid class. Phosphatidylcholine (PC) and phosphatidylethanolamine (PE), their derivates lyso-PC (LPC) and lyso-PE (LPE) and associated ether phospholipids (PC(O-), LPC(O-), PE(O-), and LPE(O-)). Total levels of other phospholipid classes are shown in Suppl. Fig. 1. Total phospholipid levels are defined as the summation of the relative abundance of all identified phospholipid species of the same class normalised to the corresponding internal standard, assuming identical response with respect to internal standard. Figures in b to d are shown as box-and-whisker plots, with floral-white plots indicating the control group (*n* = 7), white plots indicating the ACBD5-deficient cell line (*n* = 3; technical replicates), light gray plots the ACOX1-deficient group (*n* = 7), dim gray plots the ABCD1-deficient group (*n* = 7), and dark gray plots the DBP-deficient group (*n* = 6). One-way ANOVA with Dunnett’s multiple comparisons test was performed to determine significant differences between the groups, when compared to the healthy control group (* *p*-value <0.05; ** *p*-value <0.01; *** *p*-value <0.001; **** *p*-value <0.0001)

To obtain an overview of the general clustering of samples based on their phospholipid composition profiles, we used partial least squares regression discriminant analysis (PLS-DA) (Gromski et al 2015). As shown in the score plot (Fig. 2A), control and ALD fibroblasts were clustered together along the axis of the first component (X-variate), which explains the largest variability in the data. DBP- and ACOX1-deficient fibroblasts, on the other hand, were clearly separated in a different cluster, indicating large differences in phospholipid profiles when compared to the other groups. Samples from the ACBD5-deficient cell line were located between the two clusters. The phospholipid species contributing the most to the clustering along the first component were phosphatidylcholine (PC), lyso-PC (LPC), phosphatidylethanolamine (PE), the corresponding ether phospholipids (PC(O-) and PE(O-), respectively, and bis(monoacylglycero)phosphate (BMP) (Suppl. Table B). Along the axis of the second component (Y-variate), the samples from the ACBD5-deficient cell line were separated from control, ACOX1- and DBP-deficient fibroblasts but clustered together with the ALD cells (Fig. 2A).

**Fig. 3:**
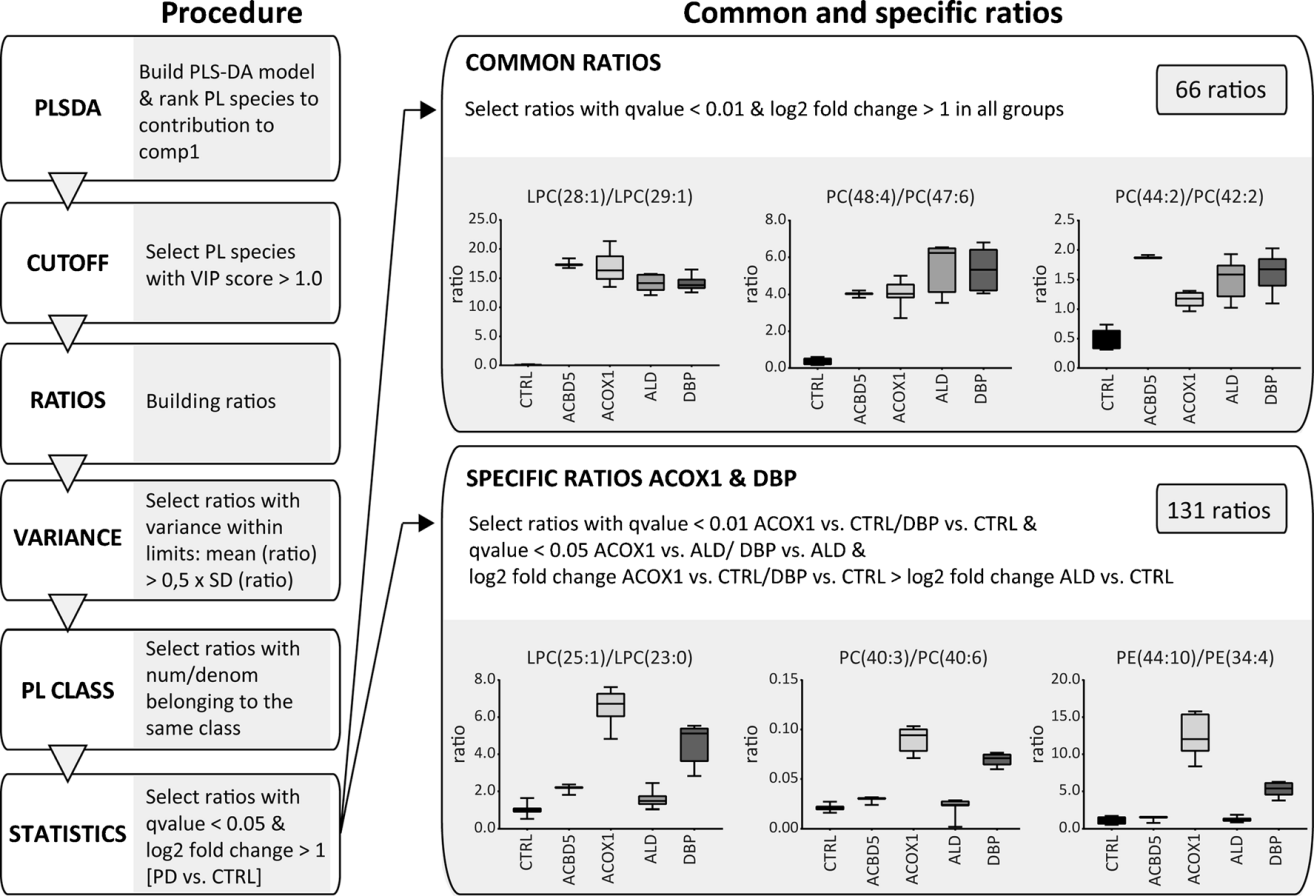
Common and specific ratios for fibroblasts from PED patients. Flowchart for data processing, filtering and selection of common and specific ratios per group. Left panel: processing procedure to obtain ratios. Right panel: statistical restrictions for the different conditions to obtain common ratios for all PED groups when compared to control samples, and specific ratios for affected enzymes located inside the peroxisome (ACOX1- and DBP deficiency) when compared to disorders with the affected enzymes outside the peroxisome (ALD, ACBD5 deficiency) and controls. For both common and specific ratios, three box plots are presented as examples. All ratios are listed in Suppl. Table D. Ratios are shown as box-and-whisker plots, and ratio values are based on the relative abundance of the phospholipid species after normalisation to the corresponding internal standards

In general, we observed increased levels of phospholipid species containing VLCFAs (species with more than 22 carbon atoms in one fatty acid side chain or more than 40 carbon atoms in combined side chains) in ACOX1- and DBP-deficient fibroblasts when compared to healthy control cells, especially in phospholipid classes such as PE, PI, BMP, and LPC (Fig. 2B; Suppl. Table A). This corresponded well with the classic measurement of total VLCFA levels by GC-MS, both the absolute C26:0 levels and the C26:0/C22:0 ratio mirrored the corresponding lipidomic parameters. (Fig. 2C). Furthermore, we found decreased levels in a variety of phospholipid species with shorter fatty acyl chain length (< C40). Similar to findings in Zellweger spectrum disorders (ZSD) fibroblasts (Herzog et al 2016), we observed a shift in phospholipid species with long-chain fatty acids towards species with VLCFAs in several phospholipid classes, especially in PC, BMP, PE, and PA (Suppl. Table A). Similarly, we also found increased levels of phospholipid species containing VLCFAs, and increased total levels of VLCFAs in ALD and ACBD5-deficient fibroblasts (Fig. 3b, c).

Notably, total levels of the mitochondrial phospholipid cardiolipin (CL) were significantly decreased in PED fibroblasts when compared to control cells (Suppl. Fig. 1).

### Decreased levels of ether phospholipids in ACOX1 and DBP deficiency

As shown in Fig. 2D, we found that various individual ether phospholipids species were significantly decreased in ACOX1- and DBP-deficient cells, also reflected by significantly decreased total levels of PC-ether phospholipids (Fig. 2D). The total levels of lyso-PC(O-) (LPC(O-)), a class of ether phospholipids containing only one fatty alcohol chain, were significantly decreased in ACOX1-deficient fibroblasts (Fig. 2D). Similarly, we observed significantly decreased levels of all ether phospholipids in the ACBD5-deficient cell line. We investigated whether all types of PC-ether phospholipids were decreased, or whether the decrease was specific for plasmalogens (ether-lipids with an O-(1-alkenyl) or vinyl-ether bond, also called plasmenyl-lipids) or plasmanyl-lipids (ether-lipids with an O-(1-alkyl)) (Fig. 1b). Plasmanyl-lipids are stable in acidic conditions, whereas the vinyl ether bond of plasmenyl-lipids is readily hydrolysed in these conditions (Kayganich and Murphy 1992). We therefore treated pooled PED fibroblast samples with hydrochloric acid and analyzed the ether phospholipids. Our results show that both plasmanyl- and plasmenyl-PC-ether phospholipids were decreased in all PED groups (Suppl. Fig. 2, Suppl. Table C).

### Decrease of phospholipid species containing docosahexaenoic acid

Interestingly, a number of phospholipid species which contained PUFAs were decreased in ACOX1- and DBP-deficient fibroblasts, but not in ALD cells. We fragmented a number of these phospholipid species, and detected that especially species containing eicosapentaenoic acid (C20:5ω-3, EPA) and docosahexaenoic acid (C22:6ω-3, DHA) were decreased in ACOX1- and DBP-deficient cells when compared to control fibroblasts (Suppl. Table C).

### Phospholipid ratios identified specific patterns in different PED disorders

To identify ratios of specific phospholipid species that discriminate between the different PED groups, we used the PLS-DA models for each PED group versus the control group based on an algorithm we recently developed (Herzog et al 2016), with minor changes (Fig. 3, Suppl. Fig. 3; Suppl. Table B). In brief, we ranked all phospholipid species based on the variable importance in projection (VIP) score in the model, and selected phospholipid species with a VIP score of >1.0. We filtered the list of ratios to eliminate features with high variance within a group and only allowed ratios with both numerator and denominator belonging to the same phospholipid class. The final set of ratios contained all features that fitted a cut-off requirement including both q-values <0.05 and a log2 fold change of >1 when the PED group was compared to the healthy control group (Fig. 3). Based on the PLS-DA models for each group, the majority of phospholipid ratios consisted of PC and PE species, including the corresponding ether phospholipid species, and a number of LPC and sphingomyelin species (Fig. 3; Suppl. Table B). Since the samples from the single ACBD5-deficient cells were technical replicates only, we excluded these samples from the ratio procedure.

Next, we identified common ratios that would discriminate all PED samples when compared to healthy control samples, and specific ratios for one PED group when compared to the other groups and samples from healthy controls. By refining the statistical restrictions, we identified 66 common ratios that were shared among all PED groups, which mainly consisted of PC-, LPC-, and PE species containing VLCFAs, such as LPC(28:1)/LPC(29:1) (Fig.3; Suppl. Table D). In addition, we identified 131 specific ratios that separated samples from ACOX1- and DBP-deficient fibroblasts from the ALD and control cells, including for example PC(40:3)/PC(40:6) (Fig.3; Suppl. Table D). Table 1 shows examples of the best common PED ratios and discriminative ratios for ACOX1 and DBP. We did not identify unique ratios for either ACOX1- or DBP-deficient cells. In general, mainly ratios containing PC species were present in all sets of discriminative ratios in all four groups, which mostly consisted of PC species containing VLCFAs and/or fatty acyl with odd chain numbers, along with a variety of ether phospholipids (Fig.3; Table 1; Suppl. Table D).

**Table 1:** Examples of common ratios for all PED fibroblasts and discriminative ratios for ACOX1 and DBP fibroblasts

Common ratios	Specific ratios ACOX1 + DBP
LPC(25:0)/LPC(24:0)	LPC(22:2)/LPC(16:2)
LPC(28:1)/LPC(29:1)	LPC(24:2)/LPC(22:1)
PC(43:1)/PC(42:1)	PC(39:5)/PC(36:5)
PC(45:1)/PC(44:1)	PC(40:1)/PC(40:7)
PC(45:2)/PC(44:2)	PC(40:3)/PC(40:6)
PC(46:1)/PC(44:0)	PC(41:4)/PC(40:6)
PC(46:2)/PC(44:2)	PC(43:6)/PC(40:7)
PC(47:5)/PC(46:5)	PC(44:7)/PC(40:5)
PC(48:2)/PC(44:0)	PE(42:7)/PE(32:2)
SM(d44:1)/SM(d43:1)	PE(44:10)/PE(34:4)

Ten representative examples of the best common PED ratios, and discriminative ratios for ACOX1 and DBP fibroblasts are shown. The majority of ratios consisted of PC-, LPC-, and PE species containing VLCFAs (common and specific ratios), and species containing fatty acyl with high number of unsaturations (specific ratios for ACOX1 and DBP fibroblasts. For details, see Suppl. Table D

To further evaluate the common ratios, we compared our results with the set of ratios obtained from the lipidomics study in ZSD fibroblasts with a mutation in the *PEX1* gene (Herzog et al 2016). First, we adjusted the lipidomics data of the ZSD fibroblasts according to the refined method described above (Fig. 3). The majority of common ratios we identified for the samples from PED fibroblasts were also discriminative for cells derived from ZSD cells when compared to control fibroblasts (46 out of 66 ratios; Suppl. Table E).

## Discussion

Peroxisomal disorders comprise a broad spectrum of metabolic diseases, including the PBDs and PEDs (Waterham and Ebberink 2012). The latter group includes a number of single enzyme defects affecting the β-oxidation of VLCFAs, including ALD, and ACBD5-, ACOX1-, and DBP deficiency (Waterham et al 2016). Whereas both ACOX1- and DBP deficiency resemble the clinical presentation of PBDs, other single enzyme deficiencies, including ALD, are clinically distinct (Poll-The and Gärtner 2012). The lipidomic profiles presented in this paper reflect the heterogeneity of peroxisomal single enzyme deficiencies that affect fatty acid β-oxidation. Especially, the phospholipid profiles of ACOX1- and DBP deficient fibroblasts were markedly different when compared to control cells.

In general, one of the most apparent differences between the PED fibroblasts and the control cells was the accumulation of phospholipid species containing VLCFAs. The increase of these species was especially prominent in ACOX1- and DBP-deficient cells, and was detected in the majority of phospholipid classes. We found similar results when comparing the ACBD5-deficient cell line to control cells. The levels of VLCFA-containing phospholipids determined by lipidomic analysis corresponded well with the VLCFA accumulation as determined by GC-MS. We also observed a shift within the PC and PE phospholipid species, showing increased levels of phospholipids with VLCFAs and decreased levels of phospholipid species containing (normal) long-chain FAs. The accumulation of phospholipids with VLCFAs was also found in ALD fibroblasts, but was limited to PC and PE phospholipid classes. These results are comparable to mild and severe ZSD fibroblast, in which a similar shift of long-chain to VLCFA-containing phospholipid species in the classes of PC and PE was detected (Herzog et al 2016). Similarly, in another recently published study using single cell lines for ACBD5 deficiency, ALD, and ACOX1 deficiency, PC species containing VLCFAs were increased, but in contrast to our results, PC species containing long-chain FAs were not changed in the ACOX1-deficient cell line (Yagita et al 2017).

Remarkably, we found decreased levels of ether phospholipids, including plasmalogens, notably in ACBD5-, ACOX1-, and DBP deficient fibroblasts, but also in ALD fibroblasts, albeit less severe. This finding was unexpected, because peroxisomal β-oxidation is not a priori known to be required for ether phospholipid biosynthesis. Although the underlying mechanism of this finding is not yet known at the moment, one possible mechanism could be that palmitoyl-CoA, which is required in the peroxisome to initiate ether phospholipid biosynthesis, is primarily generated from C26:0-CoA by peroxisomal β-oxidation. A more probable mechanism, however, could be a limited supply of hexadecanol in ALD, ACBD5-, ACOX1-, and DBP-deficient fibroblasts. In studies using radio-labelled hexadecanol, incorporation of the labelled fatty alcohol into ether phospholipids was normal in PED fibroblasts, indicating that acyl-DHAP supply is not disturbed in those cells (Schrakamp et al 1988). Interestingly, hexadecanol was added externally in these experiments, whereas under normal conditions, it is generated via fatty acyl reductase 1 (FAR1), a tail-anchored protein localised in the peroxisomal membrane. The accumulation of VLCFAs in PED fibroblasts may interfere with FAR1, thereby limiting the supply of hexadecanol into the peroxisome. Besides potential disturbances in the peroxisomal part of the ether phospholipid pathway, the accumulation of VLCFAs may also cause a secondary interference at later steps in the pathway of ether phospholipid biosynthesis, which occur in the endoplasmic reticulum (ER) (Fig. 1B). The exact mechanism behind decreased ether phospholipid levels in fibroblasts with a defect in peroxisomal β-oxidation will be further investigated.

In the ACBD5-deficient cell line, both PE- and PC-ether phospholipids were markedly decreased, suggesting a general defect in ether phospholipid biosynthesis, including plasmalogens. These results are in contrast to normal levels of PE-plasmalogens that were reported in another ACBD5-deficient cell line (Yagita et al 2017). Recently, two groups independently reported that ACBD5 forms a contact side between the peroxisome and the ER by a tether with vesicle-associated membrane protein–associated proteins A/B (Hua et al 2017; Costello et al 2017). Hua et al (2017) proposed that this tether transfers membrane lipids from the peroxisome to the ER and vice versa, a mechanism required for proper ether phospholipid biosynthesis. Similar to the results in the present study, Hua et al (2017) reported decreased levels of PE-plasmalogens in ACBD5-depleted cells (Hua et al 2017).

We also found decreased levels of CL in ALD-, ACBD5-, ACOX1-, and DBP-deficient fibroblasts when compared to control cells. In addition, the levels of the CL precursors PA and PG were also moderately reduced, which may point toward a more general inhibition of the cardiolipin synthesis pathway. Since cardiolipin is an indispensable membrane lipid in mitochondria (Houtkooper and Vaz 2008), our data indicate that peroxisomal dysfunction may also influence the mitochondrial membrane composition, with possible consequences for mitochondrial function. The underlying cause for these changes remains to be elucidated.

To identify specific patterns in different PED disorders, we created ratios of phospholipid species. In general, these ratios reflect the biochemical aberrations and functions of the enzymes that are affected in the different peroxisomal β-oxidation disorders: phospholipid species that accumulate in PED fibroblasts when compared to control cells versus phospholipid species that are decreased. The most prominent combinations that are present in the list of discriminative ratios contain species with VLCFAs from different phospholipid classes as numerator. A variety of ratios reflected the shift of phospholipids containing VLCFAS (numerator) for species containing shorter FA chains (denominator), especially PC species. Furthermore, ratios with PC-ether phospholipids as denominator reflected the reduction of these species in the PED cells. Finally, a variety of phospholipid species containing PUFAs was decreased in ACOX1- and DBP deficient fibroblasts. Some of these species contained DHA, a fatty acid that is formed from tetracosahexaenoic acid (C24:6ω-3) by peroxisomal β-oxidation, and which is known to be decreased in patients with ACOX1- and DBP deficiency (Ferdinandusse et al 2001). These phospholipid species were therefore logically present as denominator in a number of ratios. Because of the similar biochemical aberrations in ACOX1- and DBP deficiency, we did not identify unique ratios for either ACOX1- or DBP-deficient fibroblasts. The use of ratios of specific lipid metabolites for the diagnosis of ZSD and ALD patients was introduced in 1984 with the ratio of hexacosanoic acid (C26:0) to docosanoic acid (C22:0) levels (Moser et al 1984). Recently, the determination of LPC(26:0) in dried blood spots has been included as a biomarker for new born screening of ALD (Hubbard et al 2009).

Using distinct ratios of phospholipid species, we show that we can discriminate between samples from PED fibroblasts, and control cells. Furthermore, the characteristic profiles identified in PED fibroblasts resulted in specific sets of ratios for fibroblasts from peroxisomal β-oxidation disorders with the affected enzymes located inside the peroxisome (ACOX1- and DBP deficiency) when compared to disorders with the affected proteins outside the peroxisome (ALD, ACBD5 deficiency). In addition, these ratios reflect the specific functions of the various enzymes, including ACOX1 and DBP, which are not only involved in peroxisomal β-oxidation of VCLFAs, but also the formation of DHA (Ferdinandusse et al 2001; Su et al 2001). Finally, the degree of VLCFA accumulation also plays a role in the different sets of ratios, with generally higher levels of VLCFAs determined in ACOX1- and DBP-deficient cells when compared to ALD and ACBD5-deficient fibroblasts. The potential diagnostic value of these ratios will be further investigated in future studies.

The substantial number of annotated metabolites detected in one experiment and the changes in the lipid profiles in patients’ fibroblasts we present in this paper demonstrate the potential of lipidomics for diagnostic approaches. Since peroxisomal disorders are known to affect plasma lipid composition, most biomarkers found in fibroblasts, or similar ones, are likely to be applicable to plasma and leucocytes from patients, such as phospholipids containing VLCFAs. Studying the lipidome in plasma and/or leucocytes using lipidomics therefore is an interesting subject for future studies.

In conclusion, we detected characteristic changes in the phospholipid profiles of cultured PED fibroblasts affecting peroxisomal β-oxidation, which reflects the heterogeneity of this group of peroxisomal disorders. Besides the accumulation of phospholipids with VLCFAs and reduced levels of species containing PUFAs, we found decreased levels of PC-ether phospholipids, in ACBD5-, ACOX1-, DBP-deficient fibroblasts. Using a specific set of phospholipid ratios, we refined the method to study the functional effects of PEDs on the phospholipid composition in the cell, which allowed us to discriminate the PEDs from control cells.

ACBD5, acyl-CoA binding domain containing protein 5; ACOX1, acyl-CoA oxidase 1; ALD, adrenoleukodystrophy; BMP, bis(monoacylglycero)phosphate; CL, cardiolipin; DBP, D-bifunctional protein; DHA, docosahexaenoic acid (C22:6ω-3); EPA, eicosapentaenoic acid (C20:5ω-3); ER, endoplasmic reticulum; LPC, lyso-phosphatidylcholine; LPE, lyso- phosphatidylethanolamine; mLCL, monolysocardiolipin; PA, phosphatidic acid; PBD, peroxisome biogenesis disorder; PC, phosphatidylcholine; PC(O-), PC ether phospholipid; PE, phosphatidylethanolamine; PE(O-), PE ether phospholipid; PED, single peroxisomal enzyme deficiency; PG, phosphatidylglycerol; PI, phosphatidylinositol; PLS-DA, partial least squares regression discriminant analysis; PS, phosphatidylserine; SLBPA, semilysobisphosphatidic acid; SM, sphingomyelin; UPLC-HRMS, ultra-high performance liquid chromatography coupled with high-resolution mass spectrometry; VIP, variable importance in projection; VLCFA, very long-chain fatty acid; ZSD, Zellweger Spectrum Disorder.

## Electronic supplementary material

ESM 1 (DOCX 1363 kb)

ESM 2 (XLSX 7915 kb)

ESM 3 (XLSX 56 kb)

ESM 4 (XLSX 16 kb)

ESM 5 (XLSX 110 kb)

ESM 6 (XLSX 22 kb)
