# Supplementary material for: Functional characterisation of peroxisomal β-oxidation disorders in fibroblasts using lipidomics
Source: J Inherit Metab Dis. 2017 Aug 28;41(3):479–87. doi: 10.1007/s10545-017-0076-9 (PMC5959964; doi:10.1007/s10545-017-0076-9)
Supplement: Supplementary file 1 — (DOCX 1363 kb) [file 10545_2017_76_MOESM1_ESM.docx]

**Supplemental Figures and Tables**

**Supplemental Figure 1.**

**
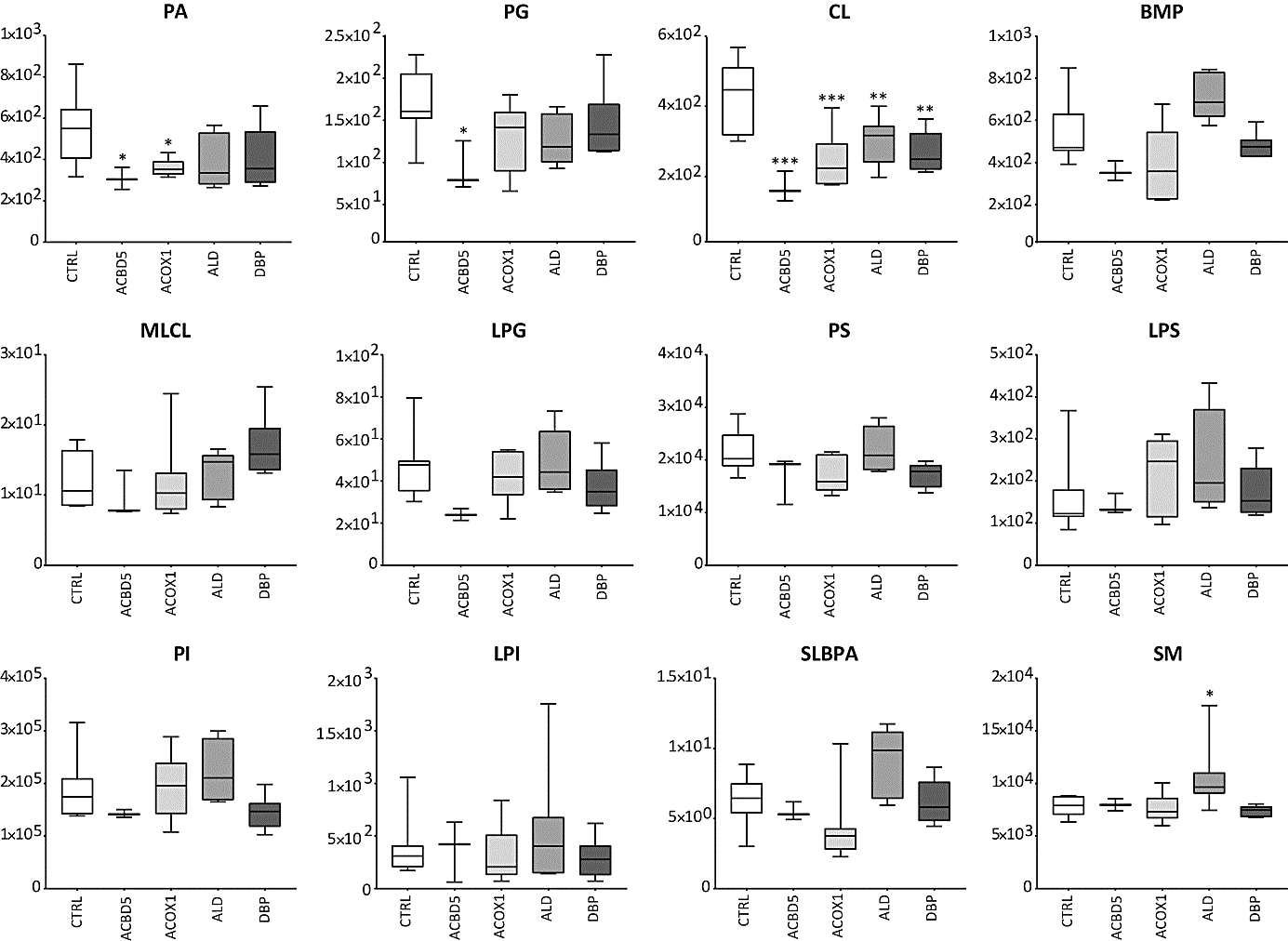
**

Additional data on total phospholipid levels, sorted by phospholipid class. Upper row: phosphatidic acid (PA), phosphatidylglycerol (PG), cardiolipin (CL), bis(monoacylglycero)phosphate (BMP). Middle row: monolyso-cardiolipin (mLCL), lyso-PG (LPG), phosphatidylserine (PS), and lyso-PS (LPS). Lower row: phosphatidylinositol (PI), lyso-PI (LPI), semilysobisphosphatidic acid (SLBPA), and sphingomyelin (SM). Total phospholipid levels are defined as the summation of the relative abundance of all identified phospholipid species of the same class normalised to the corresponding internal standard, assuming identical response with respect to internal standard (except for SLBPA, PI and LPI, because no internal standard available was available). For details, see Figure 2D.

**Supplemental Figure 2.**

**
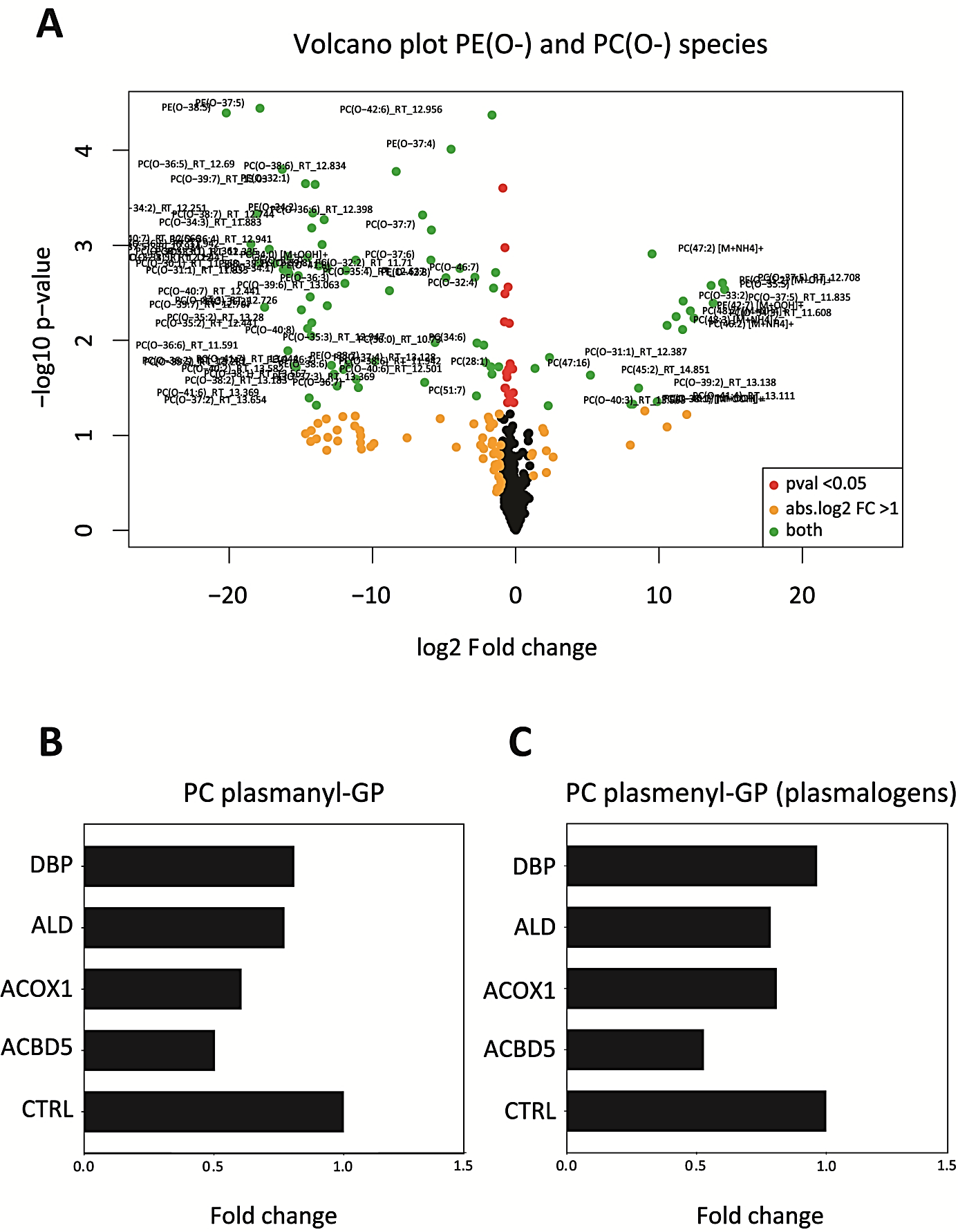
**

Identification of plasmanyl- and plasmenyl-ether phospholipids (plasmalogens) by acidification of samples. Pooled fibroblast samples per group were treated with 3 N HCl in MeOH (1:1), and incubated for 30 min at room temperature. Samples were dried under a nitrogen steam, and residues were resuspended CHCL3:MeOH=1:1. A) Volcano plot of treated versus untreated samples. Phospholipid species that were unchanged between treated and untreated samples (depicted as black dots in Volcano plot) were determined as plasmanyl-ether phospholipids, species that were decreased in treated samples when compared to untreated samples (depicted as green dots in Volcano plot, with p-value < 0.05 and log2 fold change > 1) were determined as plasmenyl-ether phospholipids (plasmalogens). B+C) Box plots of (B) plasmanyl- and (C) plasmenyl-ether phospholipids (plasmalogens) determined by acidification treatment. Values are presented as fold changes when compared to the healthy control group.

**Supplemental Figure 3.**

**
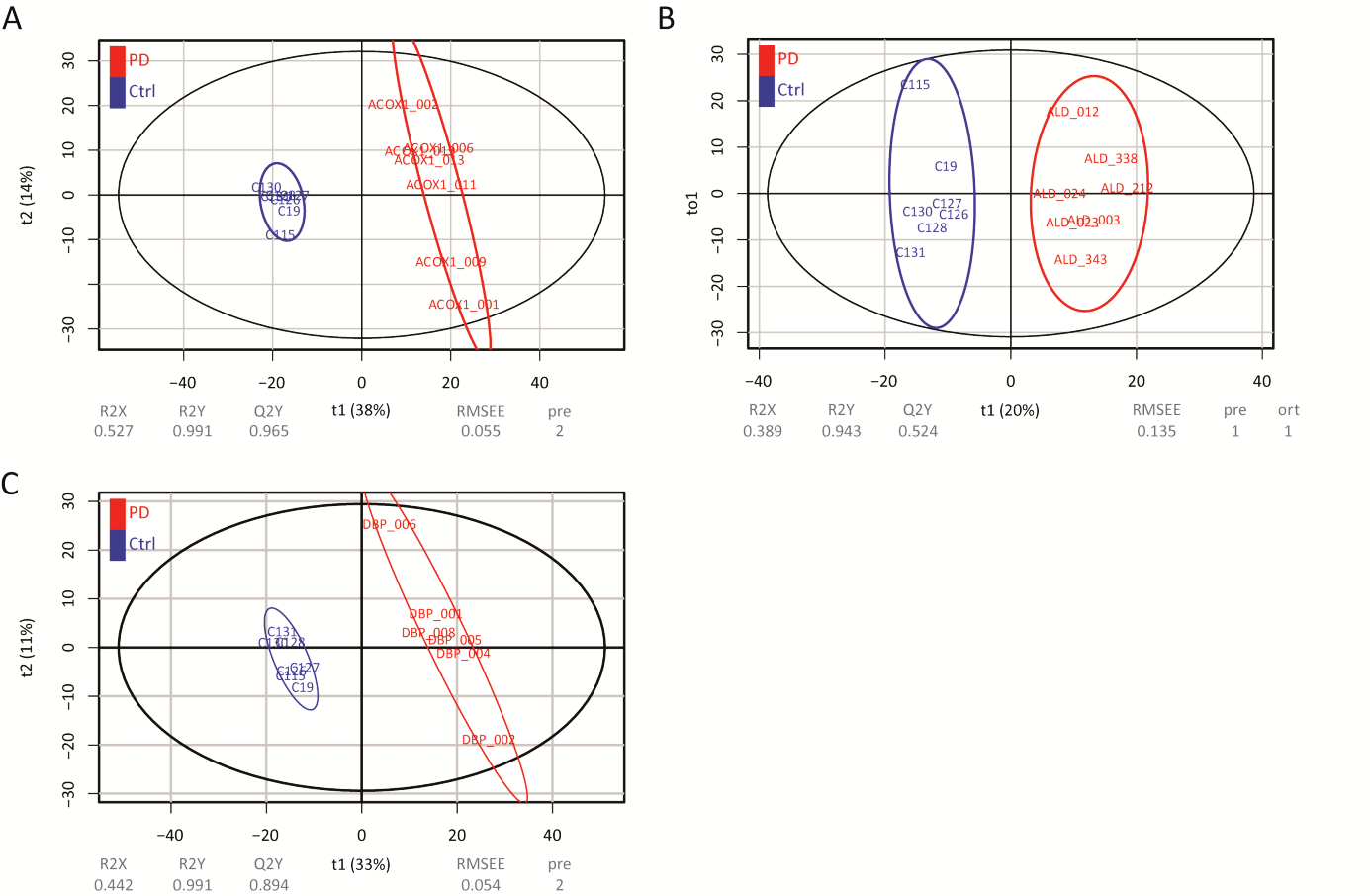
**

Score plot of the multivariate models of variations using PLS-DA for each PED group when compared to the control group. (A) ACOX1 deficiency versus control, (B) ALD versus control, and (C) DBP deficiency versus the control group. The PLS-DA model is presented in the plane of the first (X-variate) and second component (Y-variate). The percentage of variance explained by the components is indicated in parentheses. The percentage of predictor variance explained by the full model (RX2), the response variance (RY2), and the predictive performance of the model estimated by cross-validation (Q2Y) are indicated for each PLS-DA model.

**Supplemental Table A.**

List of 936 identified distinct phospholipid species, including extracted ion chromatograms (EIC’s) and box-and-whisker plots of each phospholipid species per group. The unit of the data presented is the relative abundance normalised to the corresponding internal standard, as indicated. For both PI and SLBPA, we had no internal standard available, and we therefore normalised PI species on the internal standard for PE, and SLBPA species on the internal standard for BMP, respectively. The EIC plots show the extracted ion chromatography profiles for all the samples in the dataset after retention time correction, presenting the values for the retention time (rtmed) and mass over charge ratio (mzmed) as the median of the retention times for that peak group in a sample.

**Supplemental Table B.**

List of phospholipid species ranked by the Variable Importance in Projection (VIP) score for each PLS-DA model, with a cut-off VIP score of > 1.0. PLS-DA model for (A) all groups, (B) ACOX1 deficiency versus control, (C) ALD versus control, and (D) DBP deficiency versus the control group.

**Supplemental Table C.**

List of fragmentation data obtained for several phospholipid species (A) containing fatty acids with high degree of unsaturations, which were significantly decreased in ACOX1- and DBP-deficient fibroblasts when compared to control, ABCD1- and ACBD5-deficient fibroblasts. (B) Ether phospholipid species, of which several were determined as plasmenyl-ether PLs based on acid treatment of the samples as described in supplemental Fig. 2. Identified fragmented phospholipid species were determined in pooled samples per PED group, and are listed by peak intensity. Fragmentation was performed as described in: *Hsu FF, Turk J. Electrospray ionization/tandem quadrupole mass spectrometric studies on phosphatidylcholines: the fragmentation processes. J Am Soc Mass Spectrom. 2003 Apr;14(4):352-63.*

**Supplemental Table D.**

List of A) common ratios of all PED groups when compared to healthy control samples (n=66), and B) specific ratios for affected enzymes located inside the peroxisome (ACOX1- and DBP deficiency) when compared disorders with the affected enzymes outside the peroxisome (ALD, ACBD5 deficiency) and healthy controls (n=131). For details regarding the selection procedure, see Figure 3.

**Supplemental Table E.**

List of common ratios for the samples from PED fibroblasts as shown in Fig. 3 and supplemental Table D, which were also discriminative for ZSD cells with a mutation in the *PEX1* gene when compared to control fibroblasts (as described in Herzog et al 2016) (n=46).
